# MDM2 negatively regulates the human telomerase RNA gene promoter

**DOI:** 10.1186/1471-2407-5-6

**Published:** 2005-01-18

**Authors:** Jiangqin Zhao, Alan Bilsland, Katrina Jackson, W Nicol Keith

**Affiliations:** 1 Department of Cell Physiology and Pharmacology, Faculty of Medicine and Biological Sciences, University of Leicester, Leicester, LE1 9HN, UK; 2 Centre for Oncology & Applied Pharmacology, University of Glasgow, Cancer Research UK Beatson Laboratories, Garscube Estate, Switchback Rd, Glasgow G61 1BD, UK

## Abstract

**Background:**

We have previously demonstrated that NF-Y and Sp1 interact with the human telomerase RNA (hTR) promoter and play a central role in its regulation. We have also shown that pRB activates the hTR promoter, but the mechanism of pRb directed activation is unknown. It has recently been reported that pRB induces Sp1 activity by relieving inhibition mediated by mdm2. The aim was to investigate possible roles for mdm2 in hTR promoter regulation.

**Methods:**

Chromatin immunoprecipitation was used to determine binding of mdm2 to the hTR promoter. Transfection and luciferase assays were used to investigate mdm2 repression of the promoter activity and interaction with known transcriptional modulators.

**Results:**

Here we show using chromatin immunoprecipitation that mdm2 specifically binds the hTR promoter in vivo. Transient co-transfection experiments using an hTR promoter luciferase reporter construct show that hTR promoter activity is inhibited by over-expression of mdm2 in 5637 bladder carcinoma cells (p53 and pRB negative, low mdm2). Titration of mdm2 was able to antagonise activation of hTR promoter activity mediated by pRB or Sp1 over-expression, although in the presence of pRB, mdm2 could not repress promoter activity below basal levels. Using an Sp1 binding site mutation construct we showed that mdm2 repression did not absolutely require Sp1 binding sites in the hTR promoter, suggesting the possibility of pRB/Sp1 independent mechanisms of repression. Finally, we show that NF-Y mediated transactivation of the hTR promoter was also suppressed by mdm2 in a dose-dependent manner.

**Conclusions:**

These studies suggest that mdm2 may inhibit the hTR promoter by multiple mechanisms. Mdm2 may directly repress activation by both pRB and Sp1, or activation by NF-Y. Furthermore, the ability of mdm2 to interact and interfere with components of the general transcription machinery might partly explain the general repressive effect seen here. Elucidation of new regulators affecting hTR basal promoter activity in cancer cells provides a basis for future studies aimed at improving our understanding of the differential hTR expression between normal and cancer cells.

## Background

Telomerase is a ribonucleoprotein complex that consists of an essential RNA molecule, hTR, with a template domain for telomeric DNA synthesis and of a catalytic protein, hTERT, with reverse transcriptase activity. Functional telomerase is minimally composed of both hTR and hTERT [1,2]. The transcriptional control of these two genes is a major step in the regulation of telomerase expression in human cells, with high expression of both genes detected in cancer cells relative to normal cells [3-6]. Several groups have recently reported transcriptionally targeted cancer gene therapy strategies based on the differential activities of hTR and hTERT promoters between normal and cancer cells [7-11]. Thus, investigation of the activating and repressive mechanisms of telomerase gene transcription has become an area of intense interest in cancer research.

The molecular regulation of hTR gene transcription in cancer cells remains poorly understood. The previously identified core promoter region in the hTR gene has several features utilised by the basal RNA PolII transcription machinery, including one CCAAT-box and four Sp1 sites termed Sp1.1-Sp1.4. The activity of the hTR promoter is controlled by NF-Y, Sp1 and Sp3 in bladder cancer cells in vitro and we have recently shown that an Sp1 site mutation in the hTR promoter detected in a blood sample taken from a paroxysmal nocturnal haemoglobinuria (PNH) patient can alter core promoter activity in vitro, raising the possibility that mutation might affect hTR gene transcription in hematopoietic cells *in vivo *[12-14]. Several other known transcriptional regulators, including the retinoblastoma protein pRB, are able to affect hTR transcription in the experimental setting of over-expression [12,13].

The mechanism whereby pRB activates hTR remains unknown though pRB is not known to interact directly in a specific fashion with DNA, relying instead on recruitment to genes through interaction with other transcriptional regulators including Sp1 and mdm2. The recent finding that pRB induces Sp1 activity by binding to mdm2 resulting in the physical release of Sp1 from mdm2 and enhancement of its binding to consensus sequence implies that mdm2 might inhibit promoters such as hTR that are positively regulated by pRb and Sp1 [15]. In this study, we investigated regulation of hTR reporter constructs by Sp1, pRb, NF-Y and mdm2 and performed chromatin immunoprecipitation (ChIP) assays to determine whether mdm2 plays a role in hTR regulation in the p53 and pRb negative bladder cancer cell line 5637 which also expresses relatively low levels of mdm2 [16]. We found that mdm2 interacts with the hTR promoter in vivo and that mdm2 expression can down-regulate hTR promoter activity and suppress pRb, Sp1 and NF-Y-mediated transactivation. Sp1 sites within the hTR core promoter were not absolutely required for this negative effect. These studies demonstrate that hTR transcription is dominantly repressed by mdm2 through functional, and possibly physical, interactions with the hTR promoter complex.

## Methods

### Materials and cell culture

Antibodies to Sp1, TFIIB and mdm2 were purchased from Santa Cruz Biotechnology Inc. (Santa Cruz, CA). Rabbit polyclonal antibodies directed against NF-YA, B and C were obtained from R. Mantovani (University of Milan, Milan, Italy). The 5637 cell line, originally established from the primary bladder carcinoma of a 68-year-old man in 1974, was purchased from DSMZ (No: ACC 35). 5637 cells were maintained at 37°C in 5% CO_2 _in 1640 medium supplemented with 10% foetal bovine serum, penicillin, and streptomycin.

### Plasmid construction and site-directed mutagenesis

Construction of the promoter fragment hProm867 and subcloning as an Xho I/Hind III fragment in the luciferase reporter pGL3-Basic (Promega, Madison, WI) was previously reported [17]. The reporter contains an 867 bp fragment of the hTR promoter. For generation of the Sp1 site mutation construct a two step cloning strategy was used to prevent unexpected mutations in luciferase reporter vectors; (i) an hTR 176 bp fragment (2923 wt, spanning from -107 to +69 bp) was cloned into the Xho I/Hind III sites in pCR-Script™ plasmid vector (Stratagene, La, Jolla CA), which was used as template for PCR mutagenesis using a QuikChange™ site-directed mutagenesis kit (Stratagene, La, Jolla CA) following the manufacturer's instructions. (ii) All mutation fragments were reconstructed into the Xho I/Hind III sites of pGL3-basic vectors and verified by DNA sequencing. The multiple-site mutation construct was generated in several separate PCR reactions as previously described [12].

### Transfection and dual-luciferase reporter assay

The hTR promoter plasmids containing firefly luciferase reporters were cotransfected into tumour cells with an internal *Renilla *luciferase control, pRL-SV40 (Promega) using Superfect Transfection Reagent (Qiagen) as previously described [12,13]. 5637 cells were cotransfected with 0.5 μg of expression vectors encoding wild-type NF-YA, B and C (kindly donated by Dr R. Mantovani [18]), titrations of Sp1, pRb and mdm2, 3 μg of the plasmids containing the luciferase reporter gene and 0.5 μg of pRL-SV40 plasmid for control of transfection efficiency. The total amount of DNA was kept constant at 10 μg with Salmon sperm DNA. The activity of both firefly and *Renilla *luciferase was determined 48 h later using the Dual Luciferase Assay kit (Promega). A minimum of three independent transfections were performed in duplicate and specific hTR promoter activity was normalized to protein as described elsewhere [12,13].

### Chromatin immunoprecipitation assays

Formaldehyde cross-linking and chromatin immunoprecipitation were performed as described previously [19]. In brief, 5637 cell cultures were treated with formaldehyde for 10 min followed by the addition of glycine to a final concentration of 0.125 M. Cells were then washed twice with cold PBS and were resuspended in lysis buffer (1% SDS, 10 mM EDTA, 50 mM Tris-HCI, pH 8.1) with a proteinase inhibitor. After brief sonication to fragment the DNA with an average fragment size of 500 bp, the DNA fragments crosslinked to the proteins were enriched by immunoprecipitation with specific antibodies. A "No-Ab" sample was included as a negative control for the immunoprecipitation step. After reversal of the crosslinks and DNA purification, the extent of enrichment was monitored by PCR amplification of promoters using forward and reverse primers to the hTR (5'-TACGCCCTTCTCAGTTAGGGTTAG-3' and 5'-AGCCCGCCCGAGAGAGTGAC-3') gene promoter fragments [Zhao, 2003 #9] and to the GAPDH coding region as a negative control (5'-TGAAGGTCGGAGTCAACGGATTTGGT-3' and 5'-CATGTGGGCCATGAGGTCCACCAC-3'). The PCR product was separated by agarose gel electrophoresis. The input sample was processed with the rest of the samples from the point at which the cross-links were reversed. All chromatin immunoprecipitation experiments were repeated three times and PCR analysis of individual experiments was also performed at least twice.

## Results

### Mdm2 interacts with hTR core promoter in vivo

**Figure 1:**
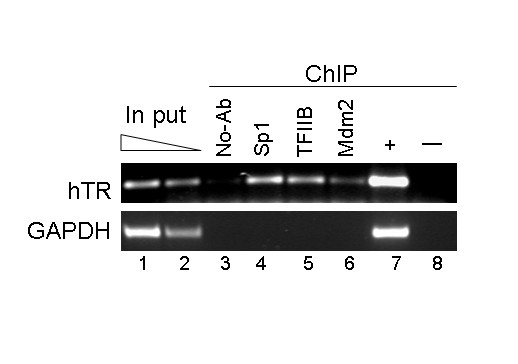
**mdm2 interacts with the hTR core promoter in vivo. **Formaldehyde cross-linked chromatin was prepared from 5637 cells and immunoprecipitated with antibodies to Sp1 (lane 4), TFIIB (lane 5) and mdm2 (lane 6), or in the absence of antibody (lane 3). PCR detection of DNA sequences immunoprecipitated with each antibody is shown in these lanes. PCR was performed with specific primers for the hTR promoter and for the GAPDH coding region as a negative control. A sample representative of the total input chromatin (input DNA lanes 1 and 2) was included in the PCR analysis. Lanes 7 and 8 show PCR positive and negative controls. Chromatin immunoprecipitation experiments were repeated three times and PCR analysis of individual experiments was also performed at least twice.

Regulation of hTR promoter activity by pRb and Sp1 suggests that mdm2 might also play a role in hTR promoter regulation. To investigate whether mdm2 protein targets the hTR promoter *in vivo *, we performed chromatin immunoprecipitation experiments using antibodies to Sp1, TFIIB and mdm2. The presence of the hTR promoter in chromatin immunoprecipitates was detected by semi-quantitative PCR. Antibodies to Sp1 and TFIIB, both of which have previously been detected at the hTR promoter in chromatin immunoprecipitation, and to mdm2 all precipitated the hTR core promoter DNA sequence but failed to precipitate the negative control GAPDH coding sequence from 5637 cells (Fig. 1). These results establish that mdm2 binds the hTR core promoter *in vivo*.

### Mdm2 represses the hTR promoter and pRb and Sp1 mediated transactivation

**Figure 2:**
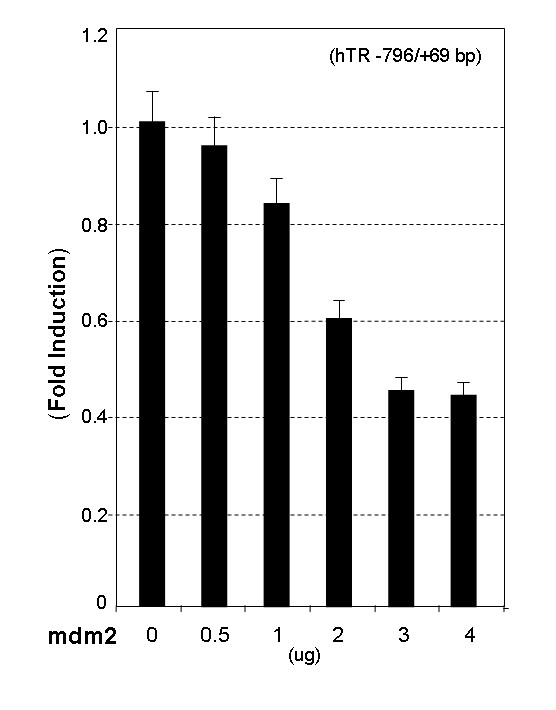
**mdm2 represses hTR promoter activity. **The pRB/p53 negative cell line 5637 were co-transfected with 1.5 μg of the pLh2023(-796/+69) construct and increasing amounts of mdm2 expression vector (from 0.5 to 4.0 μg). Total input DNA amount for transfection was adjusted with Salmon Sperm DNA to ensure a constant amount in all transfections. After 48 hour of culture, cells were harvested, and cell lysates were assayed for firefly and Renilla luciferase activity. The data is expressed as fold induction of luciferase activity relative to the promoter alone and data presented are the mean of three independent experiments performed in duplicate.

To address whether mdm2 affects the activity of the hTR promoter *in vitro*, a luciferase reporter carrying an 867 bp fragment of the hTR promoter (-798/+69) was used in transient co-transfections with a full-length mdm2 expression vector. As shown in Fig. 2, the hTR promoter was repressed by mdm2 in a dose dependent manner. Promoter activity under maximal repression by mdm2 was 42% of basal activity using 3 μg of the mdm2 expression vector.

**Figure 3:**
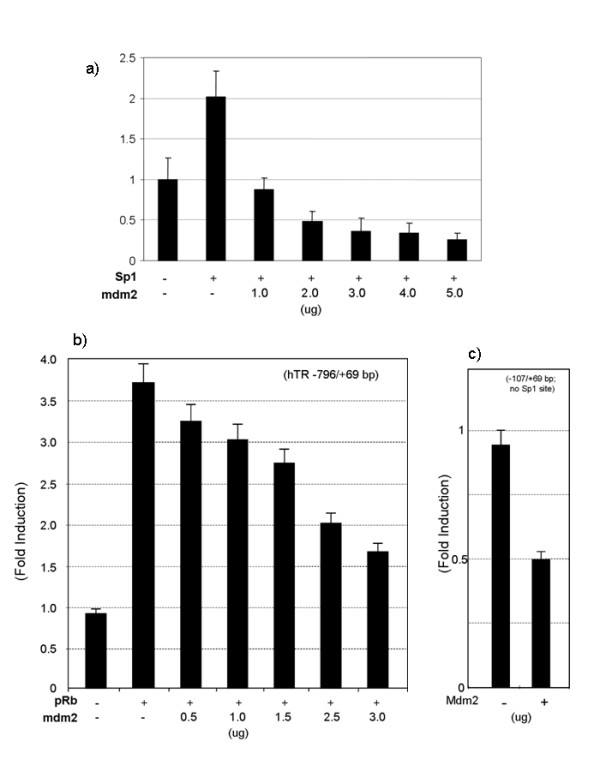
**Mdm2 represses pRb and Sp1 mediated hTR promoter transactivation a) mdm2 suppresses Sp1 activation of hTR promoter activity. **5637 cells were co-transfected with 1.5 μg of the pLh2023(-796/+69) construct, 2.0 μg of the expression vector for either Sp1 and a titration of mdm2. Total input DNA amount for transfection was adjusted with Salmon Sperm DNA to ensure a constant amount in all transfections. After 48 hour of culture, cells were harvested, and the cell lysates assayed for firefly and Renilla luciferase activity. The data is expressed as fold induction of luciferase activity relative to the promoter alone and data are the mean of three independent experiments performed in duplicate. **b) mdm2 suppresses pRB activation of hTR promoter activity **5637 cells were co-transfected with 1.5 μg of the pLh2023(-796/+69) construct, 2.0 μg of the expression vector for either pRb and a titration of mdm2. Total input DNA amount for transfection was adjusted with Salmon Sperm DNA to ensure a constant amount in all transfections. After 48 hour of culture, cells were harvested, and the cell lysates assayed for firefly and Renilla luciferase activity. The data is expressed as fold induction of luciferase activity relative to the promoter alone and data are the mean of three independent experiments performed in duplicate. **c) Sp1 sites in the hTR promoter are not essential for repression by mdm2 **5637 cells were co-transfected with 3.0 μg of the 2923 wild type or 2923 mSp1(4) mutant constructs [13], together with mdm2. After 48 hour of culture, cells were harvested, and the cell lysate were assayed for firefly and Renilla luciferase activity. The data is expressed as fold induction of luciferase activity relative to the promoter alone and the means of the three independent experiments performed in duplicate.

Mdm2 has been shown to interact with the transcription factor Sp1 *in vitro *and *in vivo *and to inhibit transactivation of Sp1-activated promoters [15,20]. We next tested whether mdm2 could interfere with Sp1 and pRb mediated transactivation of the hTR promoter. The 5637 cell line expresses low levels of mdm2 and does not express functional p53 or pRb [16]. As shown in Fig. 3A, and as previously reported, co-transfection of 5637 cells with Sp1 increased hTR promoter activity 2-fold relative to the vector control. Mdm2 overexpression was able to repress Sp1 mediated activation at all mdm2 concentrations, with Sp1 mediated induction completely abolished in the presence of 1 μg mdm2. Even in the continued presence of Sp1 overexpression, at high mdm2 concentrations promoter activity was reduced to sub-basal levels similar to those observed with mdm2 alone. Similarly, pRb overexpression led to a 3.75-fold induction of promoter activity that could be repressed in a dose dependent manner by titration of mdm2 (figure 3B). Interestingly though, at the plasmid concentrations used, even the highest concentration of mdm2 did not completely inhibit induction by pRb. Promoter activity was still induced by 1.7-fold in the presence of both pRb and 3 μg mdm2, rather than repressed to the sub-basal levels observed with Sp1/mdm2 co-expression.

To investigate whether this repression of the hTR promoter by mdm2 was entirely dependent on Sp1, we transfected a core promoter construct harboring functional mutations in all Sp1 binding sites. It should be noted the construct used here contains a shorter promoter fragment than that used throughout. Our previous study showed that mutation of all four Sp1 binding sites does not impair the activity of this hTR core promoter or its ability to be transactivated by NF-Y, but disrupts Sp1 binding and activation [13]. As shown in figure 3C, mdm2 was also able to repress this mutant, indicating that Sp1 is not essential for mdm2 mediated repression of the hTR promoter *in vitro*. Furthermore, gel shift analysis using a probe corresponding to the Sp1.3 site did not produce an mdm2 specific supershift, suggesting that mdm2 may not directly bind the hTR promoter DNA (data not shown).

### Mdm2 interferes with NF-Y-dependent activation of hTR promoter

**Figure 4:**
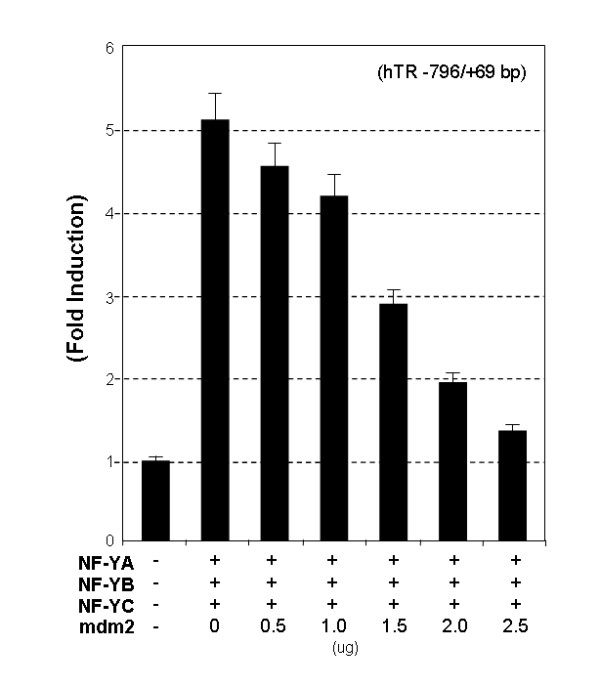
**Mdm2 inhibits NF-Y mediated hTR transactivation. **5637 cells were co-transfected with 1.5 μg of the pLh2023(-796/+69) construct, 0.5 μg of expression vectors encoding wild-type NF-YA, B and C together with increasing amounts of mdm2 expression vector (from 0 to 2.5 μg). Total input DNA amount for transfection was adjusted with Salmon Sperm DNA to ensure a constant amount in all transfections. After 48 hour of culture, cells were harvested, and the cell lysate were assayed for firefly and Renilla luciferase activity. The data is expressed as fold induction of luciferase activity relative to the promoter alone and the means of the three independent experiments performed in duplicate.

Since mdm2 can influence the basal activity of the hTR reporter without targeting its Sp1 binding sites, it is likely that at least part of the repressive effect is directed through another pathway. One alternative mechanism by which mdm2 could regulate the construct is through interfering with the NF-Y-CCAAT-box complex. Therefore, we next tested whether mdm2 could repress NF-Y function. As shown in Fig. 4, the hTR promoter is strongly stimulated (more than 5-fold induction) by co-transfection of expression vectors encoding the three NF-Y subunits, NF-YA, B and C. Titration of mdm2 again resulted in dose-dependent inhibition of promoter induction, reducing NF-Y mediated activation to 1.4-fold relative to basal levels at the highest mdm2 concentration. Thus, forced expression of mdm2 also attenuates NF-Y-activated transcription. This finding suggests that in addition to Sp1 dependent effects, down-regulation of hTR promoter activity by mdm2 may also be mediated partly by a mechanism involving inhibition of NF-Y-activated transcription.

## Discussion

The retinoblastoma gene product pRb acts as a positive regulator of hTR promoter activity by an unknown mechanism while Sp1 and NF-Y activate the hTR promoter by directly binding to DNA. Recent studies have suggested that pRb can mediate stimulatory effects at Sp1 stimulated promoters by liberating Sp1 from negative regulation by mdm2 [15,21-23]. Mdm2 regulates the activities of both p53 and pRB, and physically interacts with Sp1 to repress transcription [24-26]. Conversely, pRB interaction with mdm2 displaces Sp1 and restores Sp1 DNA binding and transactivation activity. Additionally, mdm2 protein activates the p53 pathway under stress conditions and also represses transcription directly by interaction with elements of the basal transcription machinery and their binding sites [27,28].

In this study, we used the p53 and pRb negative bladder cancer cell line 5637 to provide evidence that mdm2 interacts with the hTR core promoter *in vivo *(figure 1) and serves as a negative regulator of the hTR gene promoter in vitro (figure 2). Mdm2 opposed Sp1 and pRb directed transactivation of the hTR promoter, suggesting a plausible mechanism whereby transfected pRb may elicit its effects by opposing the action of mdm2 (figure 3). The reciprocal scenario in which mdm2 might oppose pRB cannot explain the negative effect of transfected mdm2 alone since the cells used here lack functional pRB. Rather, other mechanisms, probably including direct inhibition of Sp1 must be involved.

Mdm2 can bind directly to Sp1 and inhibit its DNA binding and can also bind to Sp1 sites at some promoters such as that of p65 [27]. Mdm2 suppressed Sp1 mediated transactivation in this study, but also had a more general repressive effect that cannot have been entirely dependent on inhibition of Sp1 since an hTR core promoter construct carrying functional mutations in all Sp1 sites was still repressed by mdm2 (figure 3C). This clearly argues against direct binding of mdm2 to those sites and implies the existence of additional repressive mechanisms.

**Figure 5:**
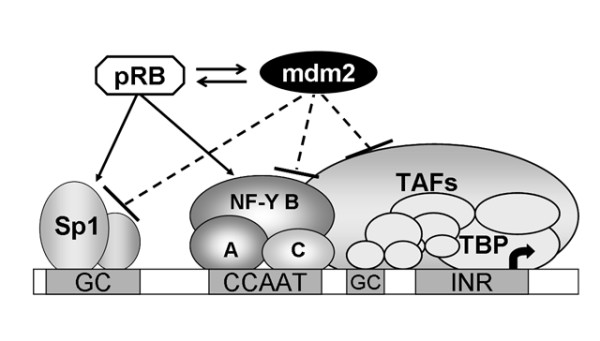
**Multiple mechanisms of the transcriptional repression of the hTR gene by mdm2. **Schematically shown are various mechanisms by which mdm2 might act on the hTR core promoter. The hTR core promoter elements include Sp1 sites (recognised by Sp1 family factors) and a CCAAT-box (recognized by NF-YA, B and C). NF-Y may facilitate the access of upstream activators (such as Sp1) to their cognate enhancer/promoter sequences, recruit TAFs components of TFIID [33] and serve to instigate the formation of a pre-initiation complex of general transcription factors and Pol II. Co-regulators such as pRB/mdm2 for modulation of signals between transcription factors and the core transcriptional machinery might act through the hTR core promoter directly or indirectly.

Here we demonstrated that activation of the hTR promoter by over-expression of all three sub-units of NF-Y could also be inhibited by mdm2 (figure 4). The CCAAT box binding protein NF-Y is a ubiquitous factor with central roles in PolII mediated transcription at numerous promoters. In cooperation with accessory proteins, NF-Y binds promoters *in vivo *before gene activation and "pre-sets" the promoter architecture allowing access by other regulatory proteins. There is also increasing evidence that NF-Y recruits multiple components of the basal PolII machinery [29-33]. Mdm2 and pRb have also been reported to interact with the basal PolII transcriptional apparatus [28,34-36]. Therefore, the possibility exists that mdm2 also inhibits this promoter through one of these other interactions although this remains to be tested (figure 5). Thus either an interaction with the basal transcriptional machinery or specific transcription factors may regulate hTR promoter activity.

## Conclusions

In conclusion, the hTR promoter is dominantly suppressed by mdm2. Mdm2 may utilise more than one mechanism to attenuate hTR promoter activity (Fig. 5). Mdm2 may directly repress activation by both pRB and Sp1, or activation by NF-Y. Furthermore, the ability of mdm2 to interact and interfere with components of the general transcription machinery might partly explain the general repressive effect seen here. Elucidation of new regulators affecting hTR basal promoter activity in cancer cells provides a basis for future studies aimed at improving our understanding of the differential hTR expression between normal and cancer cells. This will be essential for a thorough understanding of the regulation of telomerase and for advancement of telomerase directed gene therapies.

## Competing interests

The author(s) declare that they have no competing interests.

## Authors' contributions

JZ, AB and KJ carried out the transfection and genetic analysis. AB helped draft the manuscript. WNK conceived the study, participated in design and coordination and helped draft the manuscript. All authors read and approved the final manuscript.

## Pre-publication history

The pre-publication history for this paper can be accessed here:
